# Impact of medical history of severe COVID-19 infection on serum cytokine levels and prognosis in patients with sepsis: A prospective observational study

**DOI:** 10.1097/MD.0000000000046048

**Published:** 2025-11-21

**Authors:** Xinxing Tu, Jia Wang

**Affiliations:** a Department of Critical Care Medicine, People’s Hospital of Dongxihu District, Wuhan, Hubei Province, China.

**Keywords:** coagulation, COVID-19 history, cytokines, prognosis, sepsis

## Abstract

A history of severe COVID-19 may have lasting effects on immune and coagulation dysfunction, potentially influencing sepsis outcomes. This study investigates the impact of severe COVID-19 history on inflammatory markers, coagulation parameters, and prognosis in sepsis patients. This prospective observational study included 181 sepsis patients, comprising 28 with a history of severe COVID-19 infection and 153 without such a history, admitted between October 2021 and May 2023. Serum C-reactive protein (CRP), Interleukin (IL)-6, IL-1β, IL-17, and Tumor necrosis factor-α were measured by enzyme-linked immunosorbent assay (ELISA). Coagulation markers activated partial thromboplastin time (APTT), prothrombin time (PT) and D-dimer (D-D), inflammatory factor procalcitonin (PCT), and severity scores sequential organ failure assessment and acute physiology and chronic health evaluation II were assessed. Kaplan–Meier survival analysis and multivariate logistic regression were used to evaluate 28-day mortality and associated risk factors. Patients with severe COVID-19 history showed significantly higher sequential organ failure assessment and acute physiology and chronic health evaluation II scores, along with elevated APTT, PCT, IL-6, IL-1β, and IL-17 levels. CRP and tumor necrosis factor-α levels did not differ significantly. Among patients with poor prognosis, IL-6, IL-17, and APTT levels were higher, and a larger proportion had a history of severe COVID-19 (36.8% vs 5.6%). Kaplan–Meier analysis showed reduced 28-day survival in the severe COVID-19 group. Multivariate analysis identified BMI, sex, diabetes, severe COVID-19 history, PCT, D-D, and IL-17 as independent risk factors for poor outcomes. Sepsis patients with a history of severe COVID-19 exhibit more severe disease, immune dysregulation, coagulation abnormalities, and increased mortality, indicating long-term detrimental effects of prior infection on sepsis prognosis.

## 1. Introduction

Sepsis is a severe infection-related disease with high global incidence and mortality rates. Sepsis is typically caused by microbial pathogens, including bacteria, fungi, and viruses, which trigger a dysregulated host response leading to life-threatening organ dysfunction.^[1–3]^ In 2017, global estimates reported 48.9 million sepsis cases and 11 million deaths, while hospital-treated cases reached 30 million annually, with 5.3 million fatalities.^[4,5]^

Recent studies have shown that coronavirus disease 2019 (COVID-19) infection may cause pneumonia and acute respiratory distress (ARDS), cardiovascular complications, and central nervous system involvement.^[6,7]^ Patients with severe COVID-19 infection often develop pathological and physiological changes that overlap with sepsis, including systemic inflammation and immune dysregulation.^[8,9]^ Moreover, emerging evidence suggests that a history of COVID-19 infection may have adverse effects on the prognosis of patients with other diseases.^[10]^ However, research on how severe COVID-19 history affects sepsis prognosis is scarce, particularly regarding its impact on immune dysfunction, inflammation, and recovery.

Cytokines, key intercellular messengers, are essential in regulating immunity and inflammation. Wide variations in serum cytokine levels have been implicated in the pathogenesis of COVID-19.^[11]^ In the acute phase, critically ill patients exhibit markedly elevated cytokine levels, including IL-6, IL-10, and TNF-α, which correlate with disease severity.^[12]^ Importantly, recent studies have suggested that cytokine dysregulation may persist after the acute infection, with IL-6 remaining elevated in long COVID-19 patients and being implicated in neuropsychiatric sequelae.^[13,14]^ In particular, a history of severe infections, such as COVID-19, may alter the immune response in patients who subsequently develop sepsis. Overproduction of pro-inflammatory cytokines and chemokines in these patients could potentially lead to a phenomenon known as a cytokine storm, resulting in plasma leakage, vascular hyperpermeability, and disseminated intravascular coagulation, often accompanied by multi-organ dysfunction.^[15]^ During sepsis, the body releases a large amount of cytokines, with elevated IL-1β, IL-6, IL-17, and TNF-α being characteristic findings and correlating with disease severity and mortality.^[16,17]^ Although cytokine involvement in sepsis is well-researched, how severe COVID-19 history influences the alteration of cytokines remains unclear.

In this study, we aim to investigate the impact of a history of severe COVID-19 infection on serum cytokine levels and prognosis in patients with sepsis.

## 2. Methods

### 2.1. Statement of AI tool

We used ChatGPT-4o solely for language refinement, without altering the scientific content of the manuscript.

### 2.2. Participants

This prospective observational study involved 181 sepsis patients admitted to our hospital between October 2021 and May 2023. Sepsis was diagnosed following the Sepsis-3 criteria.^[1]^ A diagnosis was made if the Sequential Organ Failure Assessment (SOFA) score increased by 2 points or more from baseline (if unknown, the baseline SOFA score was assumed to be zero), or if the quick SOFA (qSOFA) score was positive (defined as the presence of 2 or more criteria: systolic blood pressure ≤100 mm Hg, respiratory rate ≥22/min, or altered mental status). Exclusion criteria included: a history of acute or chronic infections unrelated to the current sepsis episode within the past 14 days; autoimmune diseases or current use of immunosuppressive drugs; patients undergoing immunosuppressive or immune-modulating therapies; comorbid conditions that could independently influence prognosis, such as advanced malignancies, end-stage renal disease, decompensated liver cirrhosis, or established cardiovascular diseases unrelated to sepsis. Sepsis patients were divided into a severe COVID-19 H (history) group and a non-severe COVID-19 H group based on their history of severe COVID-19 infection. A history of severe COVID-19 was defined as laboratory-confirmed SARS-CoV-2 infection with severe or critical illness according to World Health Organization interim guidelines (e.g., pneumonia with SpO_2_ <90% on room air, respiratory rate >30/min, or requiring ICU admission and/or mechanical ventilation).^[18–20]^ All patients with severe COVID-19 history had been infected between January 2020 and December 2022, prior to enrollment.

All patients were consecutively enrolled, and no patient dropped out. Initially, 213 sepsis patients were screened, and after applying the inclusion and exclusion criteria, a total of 181 patients were included in the final analysis. The ethics committee of People’s Hospital of Dongxihu District approved this study, and all participants provided informed consent.

For sample size calculation, we used the formula of n=*Z*^2^*P*(1-*P*)/*d*^2^, in which *Z* is the desired confidence level (1.960 for a 95% confidence level), *P* is the estimated proportion of patients with severe COVID-19 history, and *d* denotes the margin of error (precision). Thus, according to our preliminary experiment and experience, n=*Z*^2^*P*(1-*P*)/*d*^2^=1.960^[2]^*0.120*(1-0.120)/0.473^[2]^≈181.

### 2.3. Enzyme-linked immunosorbent assay

Fasting venous blood samples (5 mL) were taken from the antecubital vein of all patients within 3 hours of ICU admission. Serum levels of C-reactive protein (CRP), interleukin (IL)-6, IL-1β, IL-17, and tumor necrosis factor (TNF)-α were measured using ELISA. The serum samples were centrifuged at 2000 g for 15 minutes and analyzed with commercially available kits (IL-17 MBS2019491, 15.6-1000 pg/mL; IL-6 MBS2019894, 0.78–50 pg/mL; IL-1β MBS3803011; TNF-α MBS824943, 15.6-1000 pg/mL; CRP MBS8123937; MyBioSource, California).

### 2.4. Data collection

Demographic and clinical data, including age, BMI, gender, comorbidities (hypertension, diabetes), and site of infection, were collected. Blood samples were analyzed using an automated biochemistry analyzer (Hitachi 7600, Hitachi Corporation, Japan). Serum was used for albumin (ALB), procalcitonin (PCT), and creatinine (Cr) measurements, while plasma was used for coagulation tests, including activated partial thromboplastin time (APTT), prothrombin time (PT), and D-dimer (D-D). Additionally, within 24 hours of intensive care unit (ICU) admission, the patients’ SOFA score and acute physiology and chronic health evaluation (APACHE) II score were calculated. Furthermore, all patients were followed up for 28 days, and those who died during the follow-up period were defined as having a poor prognosis.

### 2.5. Statistical analysis

All statistical analyses were conducted using SPSS 22.0 (IBM, Chicago), except for the Kaplan–Meier survival analysis and log-rank test, which were performed using GraphPad Prism 10.0. Data normality was assessed using the Kolmogorov-Smirnov test and the result is shown in Table S1, Supplemental Digital Content, https://links.lww.com/MD/Q656. Continuous variables were expressed as mean ± standard deviation for normally distributed data, and as median with interquartile range (Q1–Q3) for non-normally distributed data. Group comparisons for continuous variables were conducted using the Mann–Whitney *U* test for non-normally distributed data and Student *t*-test for normally distributed data. Categorical variables were compared using the Chi-square test, with degrees of freedom equal to 1 for all 2 × 2 contingency tables. Independent-samples *t*-tests were performed using SPSS (Chicago), which by default applies Levene test for variance homogeneity and provides both standard and Welch-corrected results. When variances were unequal, the Welch-corrected result was reported. To identify independent risk factors for poor prognosis, multivariate logistic regression analysis was conducted. In logistic regression, the reference categories were set as follows: male for sex, absence of diabetes and hypertension, and history of severe COVID-19 infection for COVID-19 H. Nagelkerke R^2^ was used to evaluate the goodness of fit, and variance inflation factor (VIF) calculations were performed to assess multicollinearity. The Box-Tidwell test was used to assess the linearity between continuous predictors and the logit of the outcome, and variables that violated this assumption were converted into binary variables based on their median values. Casewise diagnostics were conducted to detect extreme outliers. Model performance was assessed using the classification table (cutoff value = 0.5), which reported the overall accuracy along with class-specific prediction accuracy. A significance level of *P* *<*.05 was considered statistically significant for all analyses.

## 3. Results

### 3.1. Clinical characteristics of all participants

A comparison was made between the demographic and clinical data of the severe COVID-19 H group (n = 28) and non-severe COVID-19 group H (n = 153). As shown in Table 1, the 2 groups of patients showed no significant difference in age, BMI, sex, as well as frequency of hypertension and diabetes.

**Table 1:** Demographic and clinical data of all subjects.

Variable	Severe COVID-19 H, n = 28	Non-severe COVID-19 H, n = 153	*Z* or *χ^2^*	*P*
Age (year)*	50.5 (45–61.75)	57 (45–64)	−0.954	.340
BMI (kg/m^2^)*	23.66 (20.37–25.35)	23.09 (20.83–25.09)	<0.001	1.000
Sex, female (%)†	17 (60.71)	87 (56.86)	0.306	.580
Hypertension, n (%)†	18 (64.29)	87 (56.86)	1.156	.282
Diabetes, n (%)†	9 (32.14)	57 (37.25)	0.576	.448

The Kolmogorov–Smirnov test was used to assess data normality.

BMI = body mass index.

* Continuous variables were expressed as median (Q1–Q3) for non-normally distributed data and Mann–Whitney *U* test was applied for comparisons between the 2 groups.

† The Chi-square test was used for categorical variables.

### 3.2. Severity in sepsis patients with severe COVID-19 history

A comparison of disease severity between the severe COVID-19 H group and the non-severe COVID-19 H group is presented in Table 2. There were no significant differences in serum Cr or ALB levels between the 2 groups. However, sepsis patients with a history of severe COVID-19 exhibited significantly higher SOFA and APACHE II scores compared to those without severe COVID-19 history (*P* *<*.001 and *P* *=* .002, respectively).

**Table 2:** Disease severity between Severe COVID-19 H and Non-severe COVID-19 H patients.

Variable	Severe COVID-19 H (n = 28)	Non-severe COVID-19 H (n = 153)	*Z*	*P*
Cr (μmol/L)*	69.38 (60.33–78.61)	74.14 (60.73–90.62)	−1.412	.158
ALB (g/L)*	30.39 (26.94–32.93)	29.88 (27.40–32.39)	−0.194	.846
SOFA*	11.50 (10–13)	10 (9–12)	−4.288	<.001
APACHE II*	29.50 (25–34)	24 (20–30)	−3.172	.002

The Kolmogorov–Smirnov test was used to assess data normality.

ALB = albumin, APACHE II = acute physiology and chronic health evaluation II, Cr = creatinine, SOFA = sequential organ failure assessment.

* Continuous variables were expressed as median (Q1–Q3) for non-normally distributed data. Mann–Whitney *U* test was applied for comparisons between the 2 groups.

### 3.3. Elevated cytokines in sepsis patients with severe COVID-19 history

As shown in Table 3, sepsis patients with a history of severe COVID-19 exhibited significantly higher IL-1β, IL-6, and IL-17 levels compared to those in the non-severe COVID-19 H group (*P* *=* .002, *P* *<*.001, and *P* *<*.001, respectively). The result of Equality of Variance by Levene Test is listed in Table S2, Supplemental Digital Content, https://links.lww.com/MD/Q656. For IL-1β and IL-17, equal variances were not assumed and Welch test was used. In contrast, CRP and TNF-α levels did not differ significantly between the 2 groups (*P =* .698 and *P =* .592, respectively). Additionally, PCT levels were significantly higher in patients with severe COVID-19 history (*P* *<*.001).

**Table 3:** Cytokines between severe COVID-19 H and non-severe COVID-19 H patients.

Variable	Severe COVID-19 H (n = 28)	Non-severe COVID-19 H (n = 153)	*Z* or *t*	*P*
CRP (pg/mL)*	130.10 (115.82–145.10)	134.02 (107.11–158.91)	−0.388	.698
PCT (ng/mL)*	9.59 (7.85–10.87)	7.32 (5.16–8.82)	−5.279	<.001
TNF-α (pg/mL)*	51.99 (47.87–58.26)	50.85 (44.29–57.63)	−0.536	.592
IL-1β (pg/mL)†	17.61 ± 3.91	14.99 ± 2.78	3.381	.002
IL-6 (pg/mL)*	22.12 (20.34–24.23)	18.94 (17.84–20.90)	−4.985	<.001
IL-17 (pg/mL)†	84.90 ± 9.25	71.15 ± 9.03	7.377	<.001

The Kolmogorov–Smirnov test was used to assess data normality.

CRP = C-reactive protein, IL = interleukin, PCT = procalcitonin, TNF-α = tumor necrosis factor-α.

* Continuous variables were expressed as median (Q1–Q3) for non-normally distributed data and Mann–Whitney *U* test was applied for comparisons between the 2 groups.

† Continuous variables were expressed as mean ± SD for normally distributed data, and Student’s *t*-test was applied for comparisons between the 2 groups. For IL-1β and IL-17, equal variances were not assumed and Welch test was used.

### 3.4. Coagulation function in sepsis patients with severe COVID-19 history

As shown in Table 4, APTT levels were significantly prolonged in sepsis patients with a history of severe COVID-19 compared to those without (*P* *<*.001), suggesting a greater degree of coagulation dysfunction. However, PT and D-D levels did not show significant differences between the 2 groups (*P* *=* .097 and *P* *=* .609, respectively). These findings indicate that while APTT prolongation may be associated with severe COVID-19 history in sepsis patients, other coagulation parameters remain comparable between groups.

**Table 4:** Coagulation between severe COVID-19 H and non-severe COVID-19 H patients.

Variable	Severe COVID-19 H, n = 28	Non-severe COVID-19 H, n = 153	*Z* or *t*	*P*
APTT (s)*	43.52 (41.10–44.39)	38.80 (36.50–40.68)	−6.509	<.001
PT (s)*	15.00 (14.07–16.69)	14.65 (12.66–15.45)	−1.660	.097
D-D (μg/L)*	4.44 (3.62–5.52)	4.64 (3.70–5.55)	−0.512	.609

The Kolmogorov–Smirnov test was used to assess data normality.

APTT = activated partial thromboplastin time, D-D = D-dimer, PT = prothrombin time.

* Continuous variables were expressed as median (Q1–Q3) for non-normally distributed data. Mann–Whitney *U* test was applied for comparisons between the 2 groups.

### 3.5. Association between sepsis patients with severe COVID-19 history and prognosis

Sepsis patients were classified into poor prognosis (n = 57) and good prognosis (n = 124) groups based on 28-day mortality. As shown in Table 5, the poor prognosis group exhibited significantly higher SOFA and APACHE II scores compared to the good prognosis group (*P* *<*.001 for both), indicating greater disease severity. APTT was also significantly prolonged in the poor prognosis group (*P* *<*.001), whereas PT and D-D levels did not show significant differences between groups.

**Table 5:** Comparison of clinical characteristics of sepsis patients with good/poor prognosis.

Variable	Good prognosis group (n = 124)	Poor prognosis group (n = 57)	*Z/ t/χ^2^*	*P*
Age (year)*	58 (45.25–64)	50 (44.5–61.5)	−1.174	.240
BMI (kg/m^2^)*	23.26 (21.20–25.39)	22.13 (20.32–24.48)	−1.676	.094
Sex, female (%)†	70 (56.45)	34 (59.65)	0.210	.647
Hypertension, n (%)†	67 (54.03)	38 (66.67)	0.068	.082
Diabetes, n (%)†	46 (37.10)	20 (35.09)	0.088	.767
ALB (g/L)*	26.99 (27.38–32.05)	30.17 (27.96–32.81)	−0.663	.507
Cr (μmol/L)*	73.90 (60.95–90.62)	69.15 (57.36–87.49)	−1.544	.123
SOFA*	10 (9–11)	12 (10–12)	−5.670	<.001
APACHE II*	24 (19–30)	26 (24–33)	−4.593	<.001
APTT (s)*	38.78 (36.50–40.71)	40.68 (38.25–42.77)	−3.924	<.001
PT (s)*	14.87 (12.86–16.46)	14.61 (12.58–15.43)	−1.503	.133
D-D (μg/L)*	4.74 (3.74–5.59)	4.46 (3.56–4.94)	−1.540	.124
CRP (pg/mL)*	134.02 (103.71–158.91)	129.39 (108.60–149.30)	−0.620	.535
PCT (ng/mL)*	7.87 (6.10–8.91)	6.23 (4.92–8.86)	−1.827	.068
TNF-α (pg/mL)*	51.26 (44.10–57.63)	51.03 (47.46–58.03)	−1.142	.253
IL-1β (pg/mL)‡	15.35 ± 2.93	15.49 ± 3.53	−0.262	.794
IL-6 (pg/mL)*	19.16 (17.84–20.75)	20.70 (18.43–21.94)	−2.911	.004
IL-17 (pg/mL)‡	69.80 ± 9.03	80.84 ± 8.85	−7.682	<.001
Severe COVID-19 H, n (%)†	7 (5.6)	21 (36.8)	29.071	<.001

The Kolmogorov–Smirnov test was used to assess data normality.

ALB = albumin, APACHE II = acute physiology and chronic health evaluation II, APTT = activated partial thromboplastin time, BMI = body mass index, Cr = creatinine, CRP = C-reactive protein, D-D = D-dimer, IL = interleukin, PCT = procalcitonin, PT = prothrombin time, SOFA = sequential organ failure assessment, TNF-α = tumor necrosis factor-α.

* Continuous variables were expressed as median (Q1–Q3) for non-normally distributed data and Mann–Whitney *U* test was applied for comparisons between the 2 groups.

† The Chi-square test was used for categorical variables.

‡ Continuous variables were expressed as mean ± SD for normally distributed data, and Student’s *t*-test was applied for comparisons between the 2 groups. For IL-17, equal variances were not assumed and Welch test was used.

Among inflammatory markers, IL-6 and IL-17 levels were significantly higher in the poor prognosis group (*P* *=* .004 and *P* *<*.001, respectively), while CRP, PCT, TNF-α, and IL-1β levels did not significantly differ. The result of Equality of Variance by Levene Test is listed in Table S3, Supplemental Digital Content, https://links.lww.com/MD/Q656. For IL-17, equal variances were not assumed and Welch test was used. Notably, a higher proportion of patients in the poor prognosis group had a history of severe COVID-19 infection (36.8% vs 5.6%, *P* *<*.001), suggesting that prior severe COVID-19 may contribute to worse outcomes in sepsis patients.

**Figure 1.**
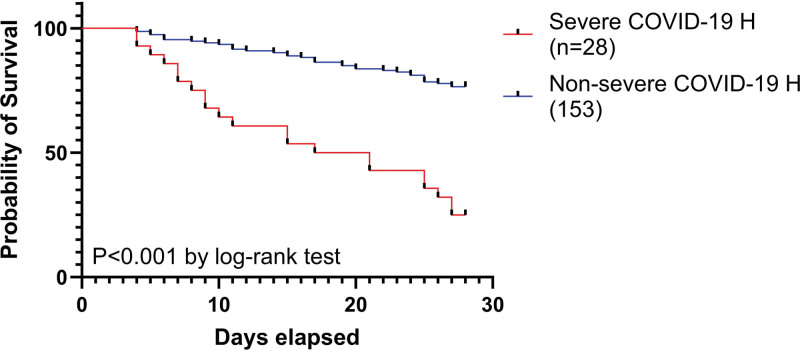
Kaplan–Meier survival curves comparing sepsis patients with and without a history of severe COVID-19.

Furthermore, Kaplan–Meier survival analysis (Fig. 1) demonstrated a significantly lower 28-day survival probability in sepsis patients with a history of severe COVID-19 compared to those without (*P* *<*.001, log-rank test).

### 3.6. Risk factors for poor prognosis in sepsis patients using logistic regression analysis

For the multivariate logistic regression analysis, we first constructed Model 1, incorporating all variables. The Nagelkerke *R*^2^ = 0.887, indicating a good model fit. Additionally, VIF calculations showed that all variables had VIF <5, suggesting no significant multicollinearity (Table S4, Supplemental Digital Content, https://links.lww.com/MD/Q656). Box-Tidwell test showed that all continuous predictors had a linear relationship with the logit of the outcome (Table S5, Supplemental Digital Content, https://links.lww.com/MD/Q656). No standardized residuals exceeded ± 3 in this model, indicating no extreme outliers. The logistic regression model correctly classified 86.7% of cases, with higher accuracy in the good prognosis group (95.2%) than in the poor prognosis group (68.4%) (Table S6, Supplemental Digital Content, https://links.lww.com/MD/Q656). The results identified BMI, sex, PCT, D-D, SOFA, APACHE II, IL-1β, and IL-17 as independent risk factors for poor prognosis in sepsis patients (Table 6).

**Table 6:** Logistic regression of risk factors for poor prognosis in sepsis patients.

Variables	Odds ratio	95% CI	*P*
Model 1			
Age	1.120	0.970–1.293	.122
BMI	0.326	0.134–0.795	.014
Sex	0.000	0.021–0.293	<.001
Hypertension	3.253	0.243–43.470	.372
Diabetes	0.112	0.008–1.654	.111
Severe COVID-19 H	0.018	0.000–2.654	.115
ALB	1.659	0.977–2.819	.061
PCT	0.191	0.074–0.491	.001
Cr	0.897	0.799–1.007	.065
APTT	1.932	0.948–3.393	.070
PT	0.771	0.324–1.833	.556
D-D	0.109	0.032–0.463	.003
SOFA	2.903	1.140–7.392	.025
APACHE II	2.025	1.362–3.012	<.001
CRP	1.016	0.952–1.083	.637
IL-6	1.868	0.928–3.762	.080
IL-1β	0.287	0.120–0.684	.005
TNF-α	1.222	0.984–1.518	.069
IL-17	1.792	1.302–2.466	<.001
Model 2			
Age	1.017	0.963–1.075	.538
BMI	0.540	0.384–0.760	<.001
Sex	0.080	0.014–0.445	.004
Hypertension	3.066	0.878–10.708	.079
Diabetes	0.192	0.050–0.748	.017
Severe COVID-19 H	0.066	0.009–0.506	.009
ALB	0.948	0.759–1.183	.635
PCT	0.370	0.226–0.604	<.001
CR	0.354	0.072–1.725	.199
APTT	1.321	0.336–5.200	.690
PT	1.045	0.313–3.487	.943
D-D	0.079	0.014–0.465	.005
CRP	0.239	0.057–1.006	.051
IL-6	1.418	0.970–2.073	.072
IL-1β	0.354	0.097–1.298	.117
TNF-α	0.535	0.148–1.934	.340
IL-17	1.279	1.142–1.433	<.001

Reference categories: sex (male), hypertension (no), diabetes (no), severe COVID-19 history (yes). For variables converted into binary form due to non-linearity, the reference category was set as lower expression (below the median, coded as 0), with higher expression (equal to or above the median, coded as 1) as the comparison group. Poor prognosis was set as the outcome group.

ALB = albumin, APACHE II = acute physiology and chronic health evaluation II, APTT = activated partial thromboplastin time, BMI = body mass index, Cr = creatinine, CRP = C-reactive protein, D-D = D-dimer, IL = interleukin, PCT = procalcitonin, PT = prothrombin time, SOFA = sequential organ failure assessment, TNF-α = tumor necrosis factor-α.

To prevent over-adjustment, SOFA and APACHE II scores were excluded in Model 2, as they are composite indices reflecting overall disease severity and could potentially confound associations between other clinical parameters and prognosis. In Model 2, the Nagelkerke *R*^2^ decreased to 0.664, with all VIF values remaining <5, confirming no multicollinearity (Table S7, Supplemental Digital Content, https://links.lww.com/MD/Q656). Box-Tidwell test results indicated that several continuous predictors, including CR, APTT, PT, D-D, CRP, IL-1β, and TNF-α, did not meet the linearity assumption with the logit (Table S8, Supplemental Digital Content, https://links.lww.com/MD/Q656). Therefore, these variables were converted into binary variables based on their median values for logistic regression analysis. Similarly, for Model 2, all standardized residuals were within the ± 3 range, confirming the absence of influential outliers. The classification table showed an overall accuracy of 86.7%, with higher accuracy in predicting good prognosis (94.4%) than poor prognosis (70.2%) (Table S9, Supplemental Digital Content, https://links.lww.com/MD/Q656). Independent risk factors for poor prognosis in sepsis patients in Model 2 included BMI, sex, diabetes, severe COVID-19 history, PCT, D-D, and IL-17.

## 4. Discussion

Due to its high mortality rate and significant economic burden, sepsis has become a global public health concern 18, 19. However, the impact of a history of severe COVID-19 on sepsis outcomes remains unclear and has not been extensively studied.^[21,22]^ Sepsis patients with a history of severe COVID-19 exhibited greater disease severity, coagulation dysfunction, and higher mortality, indicating a lasting impact of prior infection on sepsis outcomes.

The long-term impacts of SARS-CoV-2 infection have drawn global focus amid the pandemic. Numerous reports have demonstrated that SARS-CoV-2 infection can cause lung damage and directly or indirectly affect neurons, resulting in long-term pulmonary and neurological sequelae.^[23]^ Acute lung injury can lead to pulmonary fibrosis and chronic pulmonary dysfunction, impacting the quality of life and prognosis of patients.^[24,25]^ Wang et al identified COVID-19 as a poor prognostic factor in intracerebral hemorrhage.^[10]^ Heubner et al demonstrated that sepsis patients with concomitant COVID-19 had significantly higher SOFA scores upon admission, with an in-hospital mortality rate of 59%, which was notably higher than the 29% observed in non-COVID-19 patients.^[9]^ Sepsis patients with severe COVID-19 likely suffered heightened inflammation and tissue injury compared to those without. This could potentially lead to pulmonary inflammation, interstitial edema, and pulmonary tissue fibrosis, thereby increasing the risk of disease deterioration and poor prognosis in sepsis patients.^[26]^ Our findings further support this, but unlike previous studies that focused on active COVID-19 as a comorbidity, our study highlights the impact of a history of severe COVID-19 on sepsis outcomes. We observed that sepsis patients with prior severe COVID-19 had higher SOFA and APACHE II scores, increased coagulation dysfunction, and greater 28-day mortality, emphasizing the long-term effects of past infection on sepsis severity and prognosis – an aspect that has been less explored in current research.

Patients with severe COVID-19 may experience immune system dysregulation, including cytokine storms and immune dysfunction. These abnormal immune responses can lead to uncontrolled inflammatory reactions and excessive release of pro-inflammatory cytokines.^[27]^ Sepsis patients already have immune dysfunction, and the combination of the 2 can result in severe inflammatory damage and organ failure, increasing the risk of poor prognosis.^[28]^ Existing evidence has identified multiple risk factors that increase sepsis mortality, such as the presence of active cancer^[29]^ and infection originating from pulmonary, abdominal, or genitourinary sites with significant demographic variations.^[30]^ Our logistic regression analysis further complements these established predictors by highlighting BMI, sex, diabetes, severe COVID-19 history, and specific cytokines (e.g., PCT, IL-17) as additional independent risk factors, suggesting that prior severe COVID-19 may contribute to poor prognosis beyond the conventional risk profiles.

Elevated cytokine storm markers, including CRP, IL-6, and IL-10, have been linked to worsening COVID-19 cases.^[31]^ Treatment with anti-IL-17 therapy has been shown to alleviate inflammation and improve oxygenation in hospitalized patients with severe COVID-19.^[32]^ However, there has been a lack of research focusing on the impact of previous severe COVID-19 history on the expression of inflammatory factors in sepsis patients. This study uniquely reports elevated IL-6, IL-1β, and IL-17 in sepsis patients with severe COVID-19 history, consistent with the findings of studies conducted by Jekarl et al and Li et al.^[33,34]^ It has been reported that intracavity blockade of IL-17 can reduce the production of pro-inflammatory cytokines, infiltration of neutrophils, and lung injury, thereby improving the survival rate of septic mice.^[35]^ Our findings further extend this by demonstrating that sepsis patients with a history of severe COVID-19 exhibit higher IL-6, IL-1β, and IL-17 levels, along with increased disease severity and higher 28-day mortality, compared to those without such a history. This supports the notion that prior severe COVID-19 infection may contribute to a sustained inflammatory response, potentially exacerbating immune dysregulation and worsening sepsis outcomes.

Coagulation dysfunction is well-documented in sepsis patients.^[36,37]^ However, the impact of a history of severe COVID-19 on coagulation abnormalities remains unclear. In our study, APTT was significantly elevated in patients with severe COVID-19 history and those with poor outcomes, suggesting a potential link between prior infection and coagulation disturbances. Additionally, only D-D was identified as independent risk factors, further emphasizing the role of coagulation dysfunction in sepsis progression and prognosis.

## 5. Limitations

Furthermore, the limitations of our study should be acknowledged. Firstly, there was an imbalance in sample size between the groups (28 with severe COVID-19 history vs 153 without), which may affect the statistical power of comparisons and the stability of regression estimates. Secondly, we only examined a subset of clinical data and inflammatory factors, and there may be other important factors influencing prognosis that were not considered. Thirdly, the molecular mechanisms by which a history of severe COVID-19 affects inflammatory factor expression and disease prognosis in sepsis patients require further in-depth research. Besides, our study focused on inflammatory cytokines and patient prognosis rather than a comprehensive assessment of immune function. Further research is needed to elucidate the mechanisms of immune disturbances in sepsis patients with a history of severe COVID-19. Lastly, several continuous variables violated the linearity assumption in the logistic regression and were therefore converted into binary variables based on their median values to satisfy model assumptions while retaining their clinical relevance.

## 6. Conclusion

This study highlights the long-term impact of severe COVID-19 history on sepsis outcomes, demonstrating that prior infection is associated with greater disease severity, persistent immune dysfunction, coagulation abnormalities, and higher mortality. Sepsis patients with a history of severe COVID-19 exhibited elevated IL-6, IL-1β, and IL-17 levels, prolonged APTT, and increased 28-day mortality, indicating that past severe COVID-19 infection may contribute to systemic inflammation and coagulation disturbances, further worsening prognosis. These findings emphasize the need for greater awareness of the lasting effects of severe COVID-19 in critically ill patients and suggest that prior infection history should be considered when assessing sepsis risk and management strategies.

## Author contributions

**Conceptualization:** Jia Wang.

**Data curation:** Xinxing Tu, Jia Wang.

**Formal analysis:** Xinxing Tu.

**Investigation:** Xinxing Tu.

**Methodology:** Xinxing Tu.

**Project administration:** Jia Wang.

**Writing** – **original draft:** Xinxing Tu, Jia Wang.

**Writing** – **review & editing:** Jia Wang.

## Supplementary Material


