# Supplementary material for: Impact of medical history of severe COVID-19 infection on serum cytokine levels and prognosis in patients with sepsis: A prospective observational study
Source: Medicine (Baltimore). 2025 Nov 21;104(47):e46048. doi: 10.1097/MD.0000000000046048 (PMC12643713; doi:10.1097/MD.0000000000046048)
Supplement: Supplementary file 1 [file medi-104-e46048-s001.pdf]

Table S1 Normality by Kolmogorov-Smirnov test for all continuous data

	Kolmogorov-Smirnov[a]		
	Statistic	df	Sig.
AGE	.124	181	.000
BMI	.097	181	.000
ALB	.096	181	.000
PCT	.076	181	.013
CR	.092	181	.001
APTT	.099	181	.000
PT	.096	181	.000
DD	.072	181	.024
SOFA	.130	181	.000
APACHE	.115	181	.000
CRP	.113	181	.000
IL6	.082	181	.005
IL1	.048	181	.200*
TNF	.081	181	.005
IL17	.053	181	.200*

*. This is a lower bound of the true significance.

a. Lilliefors Significance Correction

Table S2 Assumption for the Student t-test in table 3 (in the main text)

Independent Samples Test										
		Levene's Test for Equality of Variances		t-test for Equality of Means						
		F	Sig.	t	df	Sig. (2-tailed)	Mean Difference	Std. Error Difference	95% Confidence Interval of the Difference	
									Lower	Upper
IL1	Equal variances assumed	8.465	0.004	4.269	179	0.000	2.61647	0.61288	1.40708	3.82586
	Equal variances not assumed			3.381	32.165	0.002	2.61647	0.77389	1.04043	4.19251

In SPSS, Welch test is used when equal variances not assumed

Independent Samples Test										
		Levene's Test for Equality of Variances		t-test for Equality of Means						
		F	Sig.	t	df	Sig. (2-tailed)	Mean Difference	Std. Error Difference	95% Confidence Interval of the Difference	
									Lower	Upper
IL17	Equal variances assumed	.147	.702	7.377	179	.000	13.75352	1.86445	10.07439	17.43266
	Equal variances not assumed			7.253	37.032	.000	13.75352	1.89630	9.91136	17.59569

In SPSS, Welch test is used when equal variances not assumed

Table S3 Assumption for the Student t-test in table 5 (in the main text)

Independent Samples Test										
		Levene's Test for Equality of Variances		t-test for Equality of Means						
		F	Sig.	t	df	Sig. (2-tailed)	Mean Difference	Std. Error Difference	95% Confidence Interval of the Difference	
									Lower	Upper
IL1	Equal variances assumed	1.686	0.196	-0.262	179	0.794	-0.13105	0.50075	-1.11919	0.85708
	Equal variances not assumed			-0.244	92.940	0.807	-0.13105	0.53611	-1.19566	0.93356

In SPSS, Welch test is used when equal variances not assumed

Independent Samples Test										
		Levene's Test for Equality of Variances		t-test for Equality of Means						
		F	Sig.	t	df	Sig. (2-tailed)	Mean Difference	Std. Error Difference	95% Confidence Interval of the Difference	
									Lower	Upper
IL17	Equal variances assumed	.134	.715	-7.682	179	.000	-11.04209	1.43747	-13.87865	-8.20553
	Equal variances not assumed			-7.741	110.904	.000	-11.04209	1.42653	-13.86889	-8.21529

In SPSS, Welch test is used when equal variances not assumed

Table S4 VIF test for model1 in logistic analysis

**Coefficients**[a]			
Model		Collinearity Statistics	
		Tolerance	VIF
1	AGE	.752	1.330
	BMI	.813	1.230
	SEX	.848	1.179
	HYPERTENSION	.751	1.332
	DIABETES	.773	1.293
	GROUPCOVID19	.372	2.689
	ALB	.751	1.331
	PCT	.607	1.647
	CR	.693	1.443
	APTT	.487	2.055
	PT	.741	1.350
	DD	.770	1.298
	SOFA	.718	1.393
	APACHE	.708	1.413
	CRP	.696	1.436
	IL6	.577	1.733
	IL1	.719	1.391
	TNF	.690	1.450
	IL17	.602	1.662
a. Dependent Variable: GROUPPROGNOSIS			

Table S5 Box-Tidwell test for model1 in logistic analysis

		B	S.E.	Wald	df	Sig.
Step 1[a]	AGE	132.358	33901.035	.000	1	.997
	BMI	-704.302	59820.320	.000	1	.991
	ALB	-1120.894	119134.597	.000	1	.992
	PCT	-387.499	21056.073	.000	1	.985
	CR	-54.892	20376.585	.000	1	.998
	APTT	1580.089	98322.335	.000	1	.987
	PT	-1787.132	218820.147	.000	1	.993
	DD	-51.962	49479.462	.000	1	.999
	SOFA	-248.982	47689.422	.000	1	.996
	APACHE	215.011	20861.441	.000	1	.992
	CRP	52.465	5916.783	.000	1	.993
	IL6	338.810	33806.190	.000	1	.992
	IL1	-205.137	13851.958	.000	1	.988
	TNF	59.756	29369.393	.000	1	.998
	IL17	-9.098	9640.520	.000	1	.999
	AGE by ln_AGE	-26.538	6763.704	.000	1	.997
	BMI by ln_BMI	170.734	14705.845	.000	1	.991
	ALB by ln_ALB	257.145	27412.612	.000	1	.993
	PCT by ln_PCT	117.206	7029.927	.000	1	.987
	CR by ln_CR	10.359	3803.777	.000	1	.998
	APTT by ln_APTT	-337.091	21004.588	.000	1	.987
	PT by ln_PT	485.165	59216.539	.000	1	.993
	DD by ln_DD	12.256	19649.949	.000	1	1.000
	SOFA by ln_SOFA	81.117	14469.673	.000	1	.996
	APACHE by ln_APACHE	-48.018	4921.087	.000	1	.992
	CRP by ln_CRP	-8.949	1018.436	.000	1	.993
	IL6 by ln_IL6	-79.023	8284.169	.000	1	.992
	IL1 by ln_IL1	54.543	3750.839	.000	1	.988
	TNF by ln_TNF	-12.587	5823.975	.000	1	.998
	IL17 by ln_IL17	2.841	1798.156	.000	1	.999
	Constant	1434.275	1087226.662	.000	1	.999

a. Variable(s) entered on step 1: AGE, BMI, ALB, PCT, CR, APTT, PT, DD, SOFA, APACHE, CRP, IL6, IL1, TNF, IL17, AGE * ln_AGE , BMI * ln_BMI , ALB * ln_ALB , PCT * ln_PCT , CR * ln_CR , APTT * ln_APTT , PT * ln_PT , DD * ln_DD , SOFA * ln_SOFA , APACHE * ln_APACHE , CRP * ln_CRP , IL6 * ln_IL6 , IL1 * ln_IL1 , TNF * ln_TNF , IL17 * ln_IL17 .

Table S6 Classification results of the logistic regression model1 predicting prognosis

**Classification Table**[a]					
	Observed		Predicted		
			GROUPPROGNOSIS		Percentage Correct
			1.00	2.00	
Step 1	GROUPPROGNOSIS	1.00	118	6	95.2
		2.00	18	39	68.4
	Overall Percentage				86.7

a. The cut value is .500

Table S7 VIF test for model2 in logistic analysis

Coefficients[a]			
Model		Collinearity Statistics	
		Tolerance	VIF
1	AGE	.806	1.241
	BMI	.777	1.286
	SEX	.786	1.273
	HYPERTENSION	.802	1.248
	DIABETES	.825	1.212
	GROUPCOVID19	.475	2.104
	ALB	.768	1.302
	PCT	.608	1.644
	CR2Group	.619	1.615
	APTT2Group	.609	1.643
	PT2Group	.785	1.274
	DD2Group	.728	1.374
	CRP2Group	.769	1.300
	IL6	.538	1.860
	IL1beta2Group	.735	1.361
	TNF2Group	.753	1.327
	IL17	.603	1.660

a. Dependent Variable: GROUPPROGNOSIS

Table S8 Box-Tidwell test for model2 in logistic analysis

Before adjusting CR, APTT, PT, D-D, CRP, IL-1β, and TNF-α						
		B	S.E.	Wald	df	Sig.
Step 1[a]	AGE	-4.453	2.591	2.955	1	.086
	BMI	-3.775	9.874	.146	1	.702
	ALB	-33.821	18.872	3.212	1	.073
	PCT	-7.669	4.027	3.626	1	.057
	CR	-6.389	2.465	6.719	1	.010
	APTT	37.894	14.885	6.481	1	.011
	PT	-64.526	24.751	6.796	1	.009
	DD	-32.861	14.355	5.240	1	.022
	CRP	2.683	.962	7.780	1	.005
	IL6	-6.749	9.704	.484	1	.487
	IL1	-14.479	4.774	9.198	1	.002
	TNF	18.736	7.526	6.197	1	.013
	IL17	-.074	3.526	.000	1	.983
	AGE by ln_AGE	.875	.514	2.895	1	.089
	BMI by ln_BMI	.777	2.403	.105	1	.746
	ALB by ln_ALB	7.753	4.326	3.212	1	.073
	PCT by ln_PCT	2.087	1.287	2.628	1	.105
	CR by ln_CR	1.200	.466	6.639	1	.010
	APTT by ln_APTT	-8.042	3.190	6.354	1	.012
	PT by ln_PT	17.517	6.738	6.758	1	.009
	DD by ln_DD	12.850	5.694	5.092	1	.024
	CRP by ln_CRP	-.464	.165	7.916	1	.005
	IL6 by ln_IL6	1.938	2.439	.631	1	.427
	IL1 by ln_IL1	3.799	1.271	8.929	1	.003
	TNF by ln_TNF	-3.781	1.520	6.188	1	.013
	IL17 by ln_IL17	.081	.660	.015	1	.903
	Constant	212.961	188.498	1.276	1	.259

a. Variable(s) entered on step 1: AGE, BMI, ALB, PCT, CR, APTT, PT, DD, CRP, IL6, IL1, TNF, IL17, AGE * ln_AGE , BMI * ln_BMI , ALB * ln_ALB , PCT * ln_PCT , CR * ln_CR , APTT * ln_APTT , PT * ln_PT , DD * ln_DD , CRP * ln_CRP , IL6 * ln_IL6 , IL1 * ln_IL1 , TNF * ln_TNF , IL17 * ln_IL17 .

After adjusting CR, APTT, PT, D-D, CRP, IL-1β, and TNF-α into categorical variables						
		B	S.E.	Wald	df	Sig.
Step 1[a]	AGE	-.862	1.550	.309	1	.578
	BMI	-1.671	6.913	.058	1	.809
	ALB	-11.848	7.252	2.669	1	.102
	PCT	-1.197	1.807	.439	1	.508
	IL6	-1.204	4.831	.062	1	.803
	IL17	.845	1.585	.284	1	.594
	AGE by ln_AGE	.170	.309	.304	1	.581
	BMI by ln_BMI	.339	1.683	.041	1	.840

	ALB by ln_ALB	2.702	1.657	2.661	1	.103
	PCT by ln_PCT	.266	.613	.188	1	.664
	IL6 by ln_IL6	.363	1.208	.090	1	.764
	IL17 by ln_IL17	-.131	.298	.194	1	.660
	Constant	88.390	72.792	1.474	1	.225

a. Variable(s) entered on step 1: AGE, BMI, ALB, PCT, IL6, IL17, AGE * ln_AGE , BMI * ln_BMI , ALB * ln_ALB , PCT * ln_PCT , IL6 * ln_IL6 , IL17 * ln_IL17 .

Table S9 Classification results of the logistic regression model1 predicting prognosis

Classification Table[a]					
	Observed		Predicted		
			GROUPPROGNOSIS		Percentage Correct
			1.00	2.00	
Step 1	GROUPPROGNOSIS	1.00	117	7	94.4
		2.00	17	40	70.2
	Overall Percentage				86.7
a. The cut value is .500					
